# Floodwater Depth Causes Different Physiological Responses During Post-flooding in Willows

**DOI:** 10.3389/fpls.2021.575090

**Published:** 2021-05-21

**Authors:** Irina Mozo, María E. Rodríguez, Silvia Monteoliva, Virginia M. C. Luquez

**Affiliations:** Instituto de Fisiología Vegetal (INFIVE), UNLP–CONICET, La Plata, Argentina

**Keywords:** *Salix matsudana* Koidz, photosynthetic rate, stomatal conductance, chlorophyll, vessels

## Abstract

Willows are widely planted in areas under risk of flooding. The physiological responses of willows to flooding have been characterized, but little is known about their responses during the post-flooding period. After the end of the stress episode, plants may modify some traits to compensate for the biomass loss during flooding. The aim of this work was to analyze the post-flooding physiological responses of willow under two different depths of stagnant floodwater. Cuttings of *Salix matsudana* NZ692 clone were planted in pots in a greenhouse. The experiment started when the plants were 2 months old with the following treatments: Control plants (watered to field capacity); plants partially flooded 10 cm above soil level (F10) and plants partially flooded 40 cm above soil level (F40). The flooding episode lasted 35 days and was followed by a recovery period of 28 days (post-flooding period). After the flooding period, height, diameter and total biomass were higher in F10, while F40 plants showed an increase in plant adventitious root production and leaf nitrogen content. During the post-flooding period, the photosynthetic rate, nitrogen, chlorophyll and soluble sugar contents were significantly higher in leaves of F40 than in Control and F10 treatments. Stomatal conductance and specific leaf area were higher in the previously flooded plants compared to Control treatment. Plants from F10 treatments showed a higher growth in height, root-to-shoot ratio, and carbon isotope discrimination than F40, while the opposite occurred for growth in diameter, vessel size and leaf area. We conclude that depth of floodwater not only causes different responses during flooding, but that its effects are also present in the post-flooding recovery period, affecting the growth and physiology of willows once the stress episode has ended. Even when flooding impacted growth negatively in F40, in the post-flooding period these plants compensated by increasing the photosynthetic rate, plant leaf area and xylem vessel size. Willows endurance to flooding is the result of both responses during flooding, and plastic responses during post-flooding.

## Introduction

The natural habitat of willows (*Salix* spp.) is floodplain areas, and they are adapted not only to endure, but also to use periodic flooding disturbances for sexual reproduction and seed dispersal (Karrenberg et al., 2002). Being a pioneer species, they have a rapid growth, and the capability of asexual reproduction through wood cuttings facilitates the development of clonal plantations with various aims, such as bioenergy, paper, timber, and wood panels, among others (Kuzovkina and Volk, 2009). In addition, willow plantations provide significant environment-protection services, like erosion control, wind and snow breaks, shelterbelts and phytoremediation (Kuzovkina and Volk, 2009).

Willows are widely planted in areas under risk of experiencing flooding episodes, and the occurrence of this stress is likely to increase due to climate change, in several areas of the world (Kreuzwieser and Rennenberg, 2014; Voesenek and Bailey-Serres, 2015). The main challenge flooding poses to plants is the energy crisis caused by the decrease in oxygen availability (Voesenek and Sasidharan, 2013; Fukao et al., 2019). Among the plant responses to flooding are changes in root-to-shoot ratio (Markus-Michalczyk et al., 2016); development of adventitious roots with aerenchyma (Li et al., 2006; Steffens and Rasmussen, 2016); a reduction in photosynthetic capacity because of stomatal and non-stomatal limitations (Kreuzwieser et al., 2002; Herrera et al., 2008a), changes in photosynthetic pigments, and carbon and nitrogen metabolism (Kreuzwieser et al., 2002; Voesenek and Bailey-Serres, 2015), and changes in leaf size, specific leaf area and leaf nitrogen content (Doffo et al., 2018; Rodríguez et al., 2018). In waterlogged seedlings, N uptake and photosynthesis were less affected in tolerant species (poplar and oak) and more in the flood sensitive beech (Kreuzwieser et al., 2002). In some species, flooding alters xylem hydraulic conductivity (Herrera et al., 2008b), and xylem vessel size and number (Copini et al., 2016; Doffo et al., 2017). These responses can also vary according to flooding duration, floodwater depth, if the water is stagnant or moving, and the age of the plant (Kozlowski, 1997; Glenz et al., 2006). In several flood-tolerant species, two extreme responses have been characterized: LOES (Low Oxygen Escape Syndrome) and LOQS (Low Oxygen Quiescence Syndrome, Voesenek and Bailey-Serres, 2015). LOES occurs in response to partial flooding and implies an increased growth response that keeps the plant above water. LOQS occurs when plants suffer a prolonged complete submergence, reducing metabolism to save energy (Voesenek and Bailey-Serres, 2015). These responses imply different signaling pathways, and also have different post-flooding responses (Voesenek and Bailey-Serres, 2015). When completely submerged, several trees, including willows, show an LOQS-type response (Iwanaga et al., 2015; Rodríguez et al., 2018).

The physiological responses of willows to partial flooding have been characterized (Li et al., 2004; Rodríguez et al., 2018), but little is known about their responses during the post-flooding period. After the end of the stress episode, plants may modify some traits to compensate for biomass loss during flooding. The aim of this work was to analyze the physiological responses to flooding and, mainly, post-flooding of willow under two different depths of stagnant floodwater. The hypotheses were: (1) Floodwater depth will have a differential impact during the post-flooding period on dry matter partitioning, photosynthetic activity, plant leaf area, vessel size and stem hydraulic conductivity; (2) Leaves expanded during and after flooding will have different morphological and biochemical characteristics that could affect their photosynthetic activity in the post-flooding period.

## Materials and Methods

### Plant Material, Growing Conditions, and Treatments

One-year-old, 20 cm long cuttings of *Salix matsudana* NZ692 clone were planted in 5 L plastic pots with a 50:50 mixture of sand and garden soil. One cutting per pot was planted on August 4, 2017. The pots were placed in a greenhouse in the city of La Plata (34° 59′ 09″ S; 57° 59′ 42″ W), with natural photoperiod and irradiance (maximum irradiance: 2,050 mmoles m^–2^ s^–1^). Until the start of the treatment, the pots were watered whenever necessary to keep them at field capacity. Sprouting occurred between August 29 and September 3, 2017. To avoid damage caused by insects, the plants were sprayed every 2 weeks with insecticide. Before the start of the treatment, cuttings were pruned and only one shoot per plant was kept, to minimize the variability in plant size induced by a different number of shoots per tree. The experiment started on October 23, 2017 with the following treatments: Control plants watered to field capacity (C); plants flooded 10 cm above soil level (F10), and plants flooded 40 cm above soil level (F40). There were 22 plants per treatment, arranged in a completely randomized design. Flooding for F10 treatment was induced by placing the pots with the trees into a sealed 10 L pot filled with tap water up to 10 cm above soil level; water was added when necessary to keep this level. For F40 treatment, plants were placed in 100 L plastic tanks filled with water that partially covered the plants 40 cm above soil level. The treatment lasted 35 days, after that, an intermediate sampling was carried out. The rest of the plants were taken out of flooding and watered daily to field capacity for 28 days (see the outline of the experiment in Supplementary Figure 1). During the post-flooding period, the plants were fertilized with 50 mL of complete Hoagland solution once a week to ensure an adequate nutrient availability.

### Growth Measurements

Total shoot height (H) was measured with a graduate stick. For each plant, the height values were plotted vs. time, and a linear function was adjusted. The growth rate in height (GRH) was determined as the slope of the adjusted straight line (Rodríguez et al., 2020). The basal diameter (D) of the shoot was measured with a digital caliper, and the growth rate in diameter (GRD) was determined as described for GRH. The dry weight of leaves, stems and roots was determined after drying them at 65∘C to constant weight. The total leaf area (TLA) was measured with a LICOR LI 3100 Area Meter (Lincoln, Nebraska, United States), discriminating between the area developed during the flooding and post-flooding periods. The specific leaf area (SLA) was determined as the ratio between the leaf area and the dry weight of the leaves expanded in each period (flooding and post-flooding).

### Gas Exchange and Carbon Isotopic Discrimination

At the beginning of the flooding treatment, a 2 cm long leaf was tagged with typing corrector (Luquez et al., 2012). This leaf was named L1 and completed its expansion during flooding. Similarly, another leaf was tagged at the beginning of the post-flooding treatment (L2). On these leaves, photosynthetic rate (A), stomatal conductance (gs) and transpiration (E) were measured with an IRGA CIRAS 2, during the post-flooding period. Measurements were performed between 10 and 13 h, with an irradiance of 1,500 mmoles m^–2^ s^–1^ and a CO_2_ concentration of 360 ppm.

After gas exchange measurements, leaf discs were frozen for chlorophyll determination (see below) and the rest of the leaf was dried at 35∘C until constant weight for carbon isotopic discrimination (Δ). To determine Δ, the leaf was grounded to powder with mortar and pestle. The determination of the carbon isotopic composition of the leaf (δC_1__3l__*eaf*_) was carried out at the INGEIS Laboratory (Instituto de Geocronología y Geología Isotópica [Geochronology and Isotope Geology Institute]) (CONICET-UBA, Buenos Aires, Argentina). The carbon isotopic composition of the air (δC_1__3a__*ir*_) was assumed to be −8‰. Δ was calculated according to Farquhar et al. (1989):

Δ=(δC-13⁢ai⁢rδC)13⁢le⁢a⁢f/(1+(δC/13⁢le⁢a⁢f1,000))(‰)

### Biochemical Determinations

Leaf discs from leaves L1 and L2 were stored at −80∘C until measurements were performed. Chlorophyll content was determined using N,N dimethylformamide according to the methods described by Inskeep and Bloom (1985).

Sugar content was determined on fully expanded leaves that were frozen and kept at -80∘C until the determinations. Insoluble and soluble reducing sugar content was determined using the Somogy Nelson method (Southgate, 1976). Frozen leaves (0.15 g) were crushed with mortar and pestle, and homogenized twice with 1 mL of 96% ethanol (v/v). The extract was centrifuged at 9,000 × g for 5 min at 4∘C. The supernatant was used for analysis of soluble reducing sugars. The pellet obtained after centrifugation was hydrolyzed with 1.5 mL of 1.1% HCl at 100∘C for 30 min. After cooling the suspension obtained, it was centrifuged at 9,000 × g for 5 min at 4∘C and the supernatant was used to analyze insoluble sugars. After the Somogy Nelson reaction, the absorbance was measured at 520 nm. Glucose was used as standard.

Total leaf nitrogen was determined on fully expanded leaves that were dried at 60∘C. The leaves were ground to powder with a hand mill and the total nitrogen content determined on 0.25 g of material according to the Kjeldahl method (Kirk, 1950).

### Hydraulic Conductivity Measurements

Hydraulic conductivity was measured in four plants of each treatment at the end of the post-flooding period, as described in Doffo et al. (2017). Measurements were performed on a segment of the basal part of the main stem. The values of the hydraulic conductivity per unit stem length (kh), the specific hydraulic conductivity per unit of xylem area (ks) and the specific hydraulic conductivity per unit leaf area (kl) were calculated according to the modified Poiseuille’s law (Cruiziat et al., 2002).

### Anatomical Analysis

The xylem anatomical analysis was carried out on a 10 cm basal segment of the main stem, just below the segment used for hydraulic conductivity measurements. At the start and end of the flooding period, small marks were made with a scalpel on the stem to injury the cambium, in order to distinguish the xylem formed in the pre-flooding, flooding and post-flooding periods (Figure 6D, Grièar et al., 2007; Monteoliva et al., 2020). The entire cross-sections of stem segments were cut using a sliding microtome, then stained with safranin (1%), and photographed with a microscope (Olympus CX30, Japan) and a digital camera (Infinity, Lumenera, Canada). The captured images were analyzed for the following parameters: vessel lumen diameter (μm), vessel area (VA, μm^2^), and vessel number (VN, mm^–2^). The analysis was performed with the image analysis software ImagePro Plus v.6.3 (Media Cybernetics, United States). The vessel’s lumen fraction (LM) was estimated as the product between vessel area and number for each period, and was expressed as the percentage of the total stem area of the plant.

**FIGURE 1:**
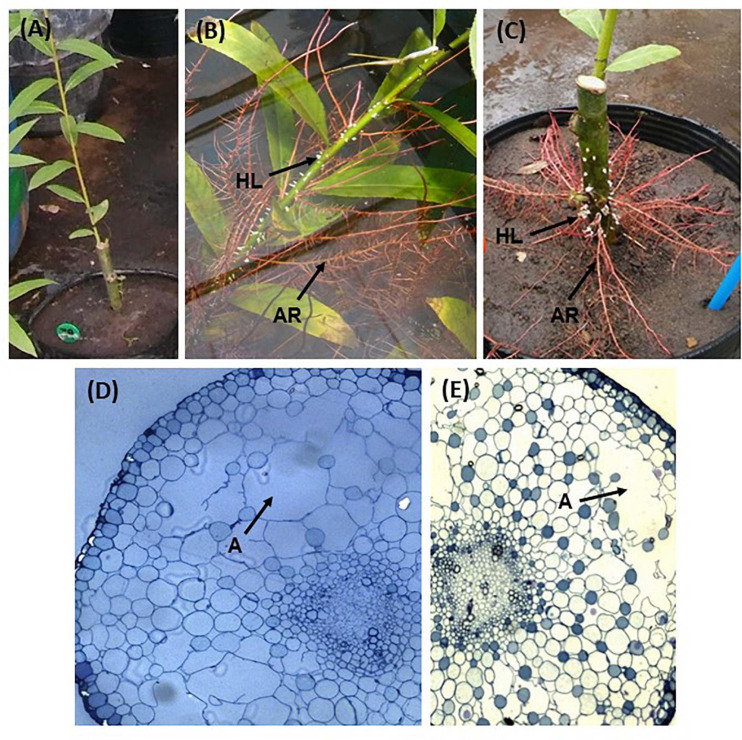
View of the stem of a control plant, without development of hypertrophied lenticels or adventitious roots **(A)**; hypertrophied lenticels (HL) and aquatic roots (AR) in plants of F40 treatment **(B)**; aquatic roots and lenticels outside water in a plant from F10 treatment **(C)**; a transversal view of F40 aquatic roots (**D**, 10×); and a transversal view of an aquatic root in F10 (**E**, 10×). **(A)** aerenchyma lacunae.

**FIGURE 2:**
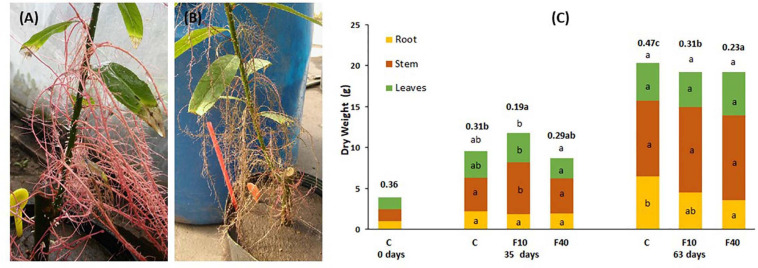
Aquatic roots of a F40 plant immediately after taken out of water **(A)** and 24 h later **(B)**. **(C)** Dry matter partitioning and root-to-shoot ratio (in bold, above the bars) at the beginning of the experiment (day 0), at the end of the flooding period (35 days), and at the end of the post-flooding recovery period (63 days). Means followed by the same letter did not differ according to Tukey’s test (*p* < 0.05, *N* = 5). The letters indicate significant differences for each compartment, total biomass (above the bar) and root-to-shoot ratio (next to this value, in bold).

**FIGURE 3:**
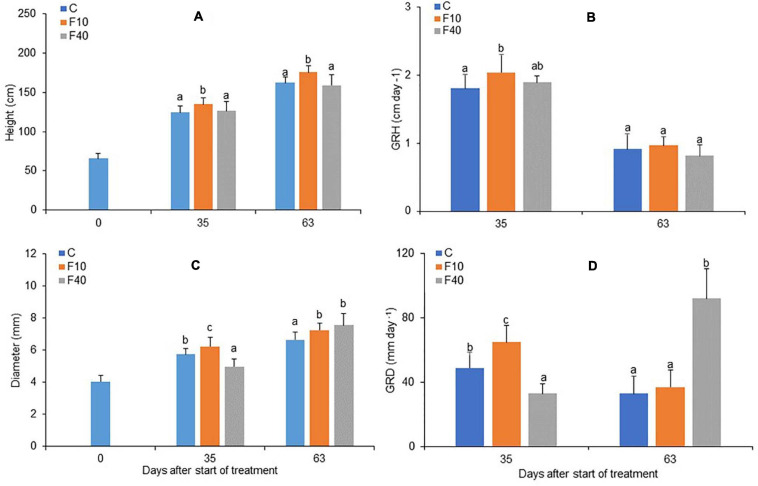
Height **(A)**, growth rate in height **(B)**, diameter **(C)** and growth rate in diameter (**D**, multiplied by 10^3^) for Control, F10 and F40 treatments at the beginning of the experiment (Day 0), at the end of the flooding period (Day 35), and at the end of the post-flooding recovery period (Day 63). Growth rates were calculated for the whole flooding and post-flooding periods. Means followed by the same letter did not differ according to Tukey’s test (*p* < 0.05, *N* = 12–22). Vertical bar: standard deviation.

**FIGURE 4:**
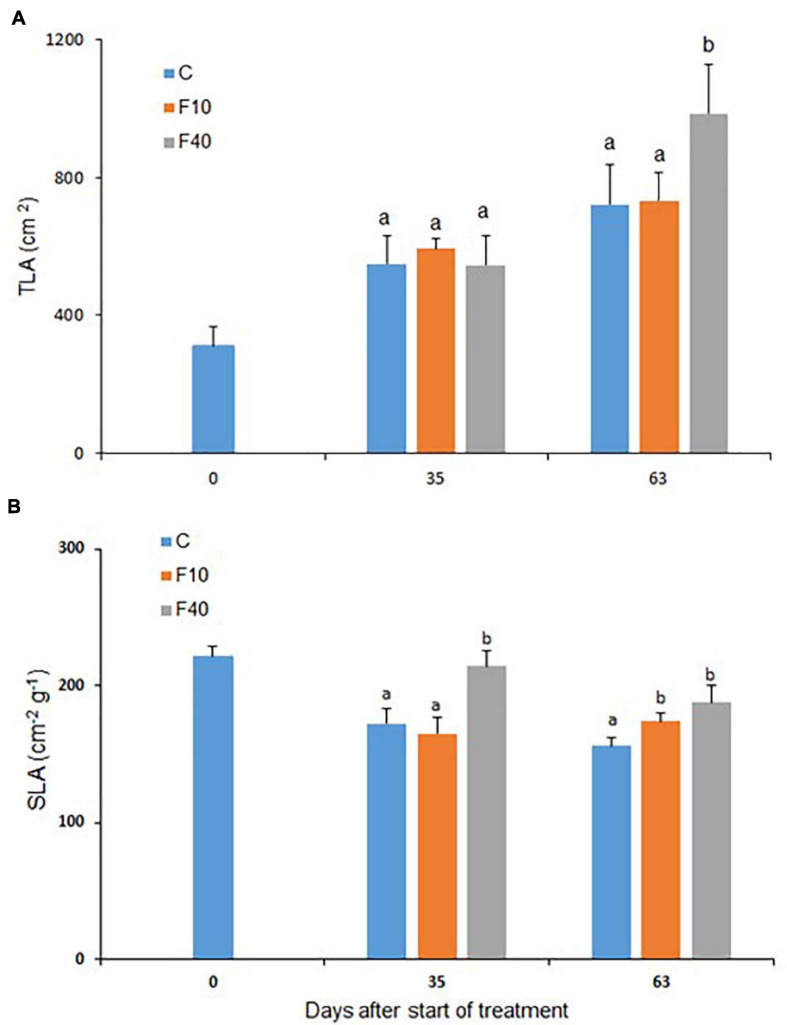
Total leaf area **(A)** and specific leaf area **(B)** for C (Control), F10, and F40 treatments at the beginning of the experiment (Day 0), at the end of the flooding period (Day 35), and at the end of the post-flooding recovery period (Day 63). Means followed by the same letter did not differ according to Tukey’s test (*p* < 0.05, *N* = 4–8). Vertical bar: standard deviation.

**FIGURE 5:**
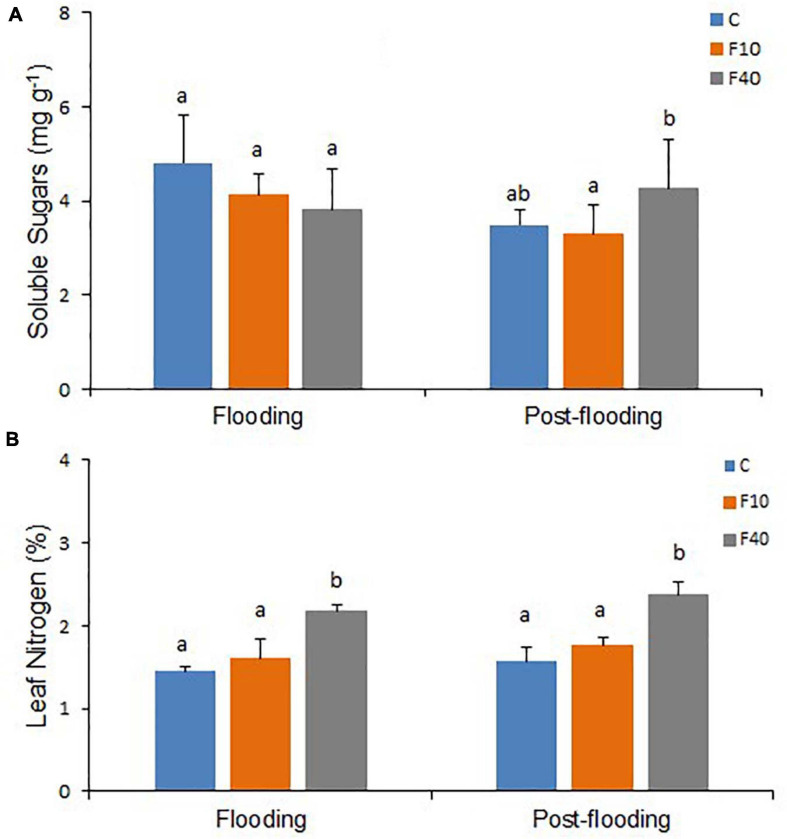
Soluble sugars content (**A**, *N* = 5) and nitrogen content (N, **B**, *N* = 3) on fully expanded leaves after 35 days of flooding, and at the end of the post-flooding recovery period (Day 63). Means followed by the same letter did not differ according to Tukey’s test (*p* < 0.05). Vertical bar: standard deviation.

**FIGURE 6:**
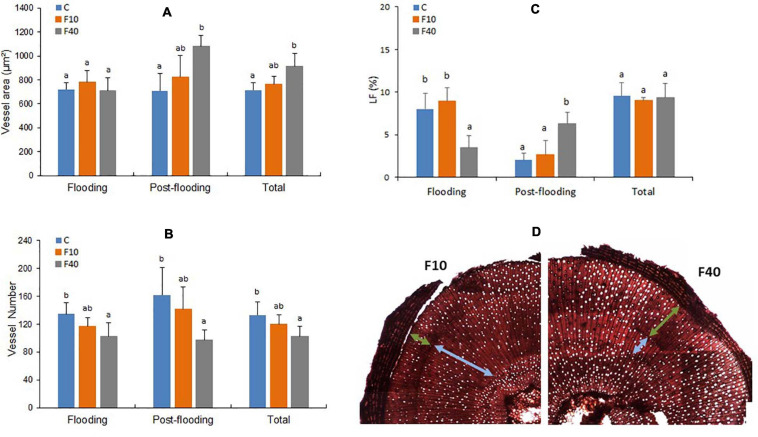
Vessel area **(A)**, vessel number **(B)**, percentage of vessel lumen fraction of the stem (LF, **C**), transversal cut of the stem with light blue arrows indicating the vessels developed during flooding, and green arrows indicating the vessels formed during post-flooding **(D)**. Total refers to the average values of all vessels in the stem (formed during pre-flooding, flooding and post-flooding). Means followed by the same letter did not differ according to Tukey’s test (*p* < 0.05, *N* = 5). Vertical bar: standard deviation.

To determine stomatal density, a fully expanded leaf was fixed in FAA (formaldehyde alcohol acetic acid 10:50:5%). The leaves were cleared for the observation of stomata, placing them for 3 days in a 50:50 mix of 5% sodium hydroxide and 5% commercial bleach. Once decolored, the material was cleaned with distilled water and kept for 48 h in a 5% chloralhydrate solution. After that, the leaves were cleaned with distilled water, stained with 1% safranin and mounted in gelatin-glycerin for observation (Arambarri, 2018). To determine stomatal density, ten fields per sample were counted at 400×.

The material for the anatomical analysis of adventitious roots and hypertrophied lenticels was fixed in FAA. Afterward, the samples were dehydrated in an alcoholic series to absolute alcohol and acetone. Samples were included in Epoxi resin for 36 h under mild vacuum at 35, 50, and 60∘C to allow polymerization. The thin sections were stained with toluidine blue and photographed at 10×.

### Statistical Analysis

The data were analyzed with a one-way ANOVA followed with a *post-hoc* mean comparison with the Tukey test (*p* < 0.05).

## Results

Flooding triggered the formation of hypertrophied lenticels (Figures 1A,B,C and Supplementary Figure 2) and adventitious roots (Figures 1B,C,E) in the stem parts covered by water. After 4 days of flooding, plants of both flooding treatments (F10 and F40) started to develop hypertrophied lenticels (Figures 1B,C and Supplementary Figure 2A). After 7 days of flooding, adventitious roots appeared in both flooding treatments (Figures 1B,C,2A and Supplementary Figure 2A). The adventitious roots developed in the stems under water; in consequence we called them aquatic roots. The aquatic roots had aerenchyma in both F10 and F40 (Figures 1D,E). At the end of the 35-day flooding period, the development of aquatic roots was profuse in F40, representing 42% of the total root biomass. After the flooding period ended, the aquatic roots dried up (Figures 2A,B).

At the end of the flooding period (Day 35, Figure 2C), F10 plants had a higher total biomass than F40 plants. The difference was due to the increase in shoot biomass (stem plus leaves). For the same reason, the root-to-shoot ratio was significantly lower in F10 compared to Control (C) and F40 treatments. The treatments showed no differences in root biomass as the result of extensive development of aquatic roots in both F10 and F40. The root-to-shoot ratio in both F10 and F40 was calculated including the aquatic root biomass.

At the end of the post-flooding period (Day 63), the treatments showed no differences in the total biomass (Figure 2C). However, there were statistically significant differences in root-to-shoot ratio, being significantly lower in F40 than in F10, and in both previously flooded plants compared to Control. The main reason is that root biomass in F40 was significantly lower than in Control plants. The reduction of root biomass in the previously flooded plants (especially F40) was due to the death of the aquatic roots immediately after the end of flooding (Figure 2B).

The height and diameter growth were differently affected in the two flooding treatments. At the end of the flooding period (Day 35), F10 showed both a significantly higher height and diameter than Control and F40 (Figures 3A,C, respectively). On the same day, F40 had lower values for diameter compared to Control and F10 treatments (Figure 3C, day 35). The growth rate in height (GRH) was significantly higher in F10 compared to Control, while F40 did not differ from the other treatments (Figure 3B, day 35). The growth rate in diameter (GRD) differed significantly among treatments, F40 had the lowest rate, while F10 had the highest (Figure 3D, day 35).

At the end of the post-flooding period (Day 63), F10 featured a higher height than Control plants, while F40 did not differ in height from Control (Figure 3A). The diameter was higher in F10 and F40 than in Control plants, but there were no differences between the previously flooded treatments (Figure 3B, day 63). GRH did not differ between the treatments in the post-flooding period (Figure 3C, day 63), while GRD was significantly higher in F40 compared to Control and F10 plants (Figure 3D, day 63).

The total leaf area (TLA, Figure 4A) did not differ between the treatments at the end of the flooding period (Day 35), but after the post-flooding period (Day 63), TLA was significantly higher in F40 than in the other treatments.

The specific leaf area (SLA, Figure 4B) on Day 35 was higher in F40 when compared to Control and F10. At the end of the post-flooding period, SLA was significantly higher in both F10 and F40 compared to Control plants.

The stomatal density did not change significantly in the abaxial surface during flooding and post-flooding periods (Table 1). At the end of the flooding period (Day 35, Table 1) the stomatal density in the adaxial surface was significantly higher in F40 than in F10. At the end of the post-flooding period (Day 63), there were no differences between the treatments for the stomatal density on any leaf surface.

**TABLE 1:** Stomatal density (stomata per mm^–2^) in fully expanded willow leaves during flooding (Day 35) and post-flooding periods (Day 63).

**Treatment**	**Day 35 (flooding)**		**Day 63 (post-flooding)**	
	***Abaxial side***	***Adaxial side***	***Abaxial side***	***Adaxial side***
Control	326 (14) a	105 (11) ab	266 (34) a	91 (6) a
F10	320 (12) a	94 (10) a	313 (35) a	111 (16) a
F40	382 (91) a	166 (42) b	334 (56) a	97 (11) a

*Days are counted after the start of the flooding treatment. Means followed by the same letter did not differ according to Tukey’s test (p < 0.05, N = 3). Between parenthesis: standard deviation.*

After 35 days of flooding, there were no differences in soluble or insoluble sugar contents of leaves in all treatments (Figure 5A and Supplementary Figure 3). However, at the end of the post-flooding period, soluble sugars content was significantly higher in the F40 plants when compared to the F10. The Leaf Nitrogen content (Figure 5B) was significantly higher in F40 than in control and F10 plants after the end of both flooding and post-flooding periods.

The leaves L1 (expanded during flooding) and L2 (expanded in the post-flooding period) were measured during the post-flooding period (Table 2). For L1, the photosynthetic rate (A) was significantly higher in F40 compared to control and F10. For L2, A was higher F40 compared to control, but it did not differ from F10. The plant photosynthetic activity was significantly higher in F40 compared to Control and F10 plants (Supplementary Figure 5). The stomatal conductance (gs) was significantly higher in F40 and F10 for L1. For L2, gs was significantly higher in F40 than Control plants, while F10 did not differ from the other treatments.

**TABLE 2:** Gas exchange and biochemical traits in a leaf expanded during flooding (L1) and a leaf expanded during post-flooding (L2).

Variable	Units	Treatment	L1	L2
Photosynthetic Rate (A)	μmol CO_2_ m^–2^ s^–1^	Control	17.2 (0.4) a	12.7 (3.5) a
		F10	18.2 (2.3) a	18.1 (2.5) ab
		F40	24.7 (3.6) b	20.7 (4) b

Stomatal Conductance (gs)	mmol H_2_O m^–2^ s^–1^	Control	356 (67) a	112 (53) a
		F10	517 (40) b	275 (95) ab
		F40	504 (107) b	315 (139) b

Carbon isotope Discrimination (Δ)	‰	Control	23.1 (0.5) a	22.9 (0.4) a
		F10	23.9 (0.6) b	23.9 (0.4) b
		F40	23.3 (0.5) ab	22.6 (0.6) a

Chlorophyll a	μg cm^–2^	Control	8.5 (1.5) a	9.6 (0.3) a
		F10	8.7 (1.1) a	9.0 (1) a
		F40	14.4 (2.4) b	15.2 (0.7) b

Chlorophyll b	μg cm^–2^	Control	2.6 (0.5) a	2.2 (0.4) a
		F10	2.6 (0.4) a	1.9 (0.3) a
		F40	4.4 (0.7) b	3.8 (0.3) b

Total Chlorophyll	μg cm^–2^	Control	11.2 (2) a	11.72 (0.6) a
		F10	11.3 (1.4) a	10.8 (1.3) a
		F40	18.7 (3.2) b	19 (0.9) b

**Determinations were carried out during post-flooding. Means followed by the same letter did not differ significantly according to Tukey’s test (p < 0.05, N = 5). Between parenthesis: standard deviation.**

Carbon isotope discrimination (D) was significantly higher for F10 compared to Control in both L1 and L2. F40 did not differ from the other treatments in L1, while for L2 was similar to control and lower than F10.

Chlorophyll content was significantly higher in F40 for both leaves, for chlorophyll a, chlorophyll b, and total chlorophyll. The chlorophyll a/b ratio did not change in any treatment (data not shown).

The results for xylem vessels diameter and area were similar, in consequence only area data are shown. The vessel area (Figure 6A, Flooding) did not differ between the treatments at the end of the flooding episode. The vessel number was significantly lower in F40 compared to Control plants, while it did no differ in F10 (Figure 6B, Flooding). In the post-flooding period, F40 produced vessels with a larger area than Control plants, while F10 did not differ from Control and F40 (Figure 6A Post-flooding). The average for all periods (Total) largely reflects the results of the post-flooding period. The number of vessels (Figure 6B) did not differ between Control and F10 plants for any period, while in F40, this trait was significantly lower than in Control plants. We also estimated the lumen fraction (LF, percentage that the vessel lumen area represents of the total stem area, Figure 6C). The percentage of vessels formed during the flooding period was similar in Control and F10 plants, while it was significantly lower in F40. During post-flooding it was the opposite: LF was significantly higher in F40. Taking together all periods (Total), there were no significant differences in LF between the treatments.

The hydraulic conductivity was measured per unit stem length (kh), per unit xylem area (ks) and per unit leaf area (kl) at the end of the post-flooding period (Day 63, Supplementary Figure 4). There were no statistically significant differences between the treatments for any of the measurements.

## Discussion

### Growth Responses to Floodwater Depth

The two flooding treatments had contrasting growth responses, not only during flooding, but also in the post-flooding period. During flooding, leaf area, biomass accumulation, height, diameter and diameter growth rate were higher in F10 than in F40. These results are similar to previous findings that, in willows, the flooding of the root system does not cause significant differences in biomass with control plants (Li et al., 2006; Rodríguez et al., 2018). The results are consistent with previous data that an increased floodwater depth enhances growth reduction (Iwanaga and Yamamoto, 2008; Markus-Michalczyk et al., 2016; Rodríguez et al., 2018). Moreover, during flooding, there was a sharp contrast in dry matter partitioning between the treatments. F10 had a higher total biomass and a lower root-to-shoot ratio than F40. While F10 behavior resembles a LOES escape strategy increasing height growth to avoid submergence (Voesenek and Bailey-Serres, 2015), F40 was neither LOES nor LOQS. Clearly, F40 plants had a reduced growth, but not a quiescent response. On the other hand, both treatments developed hypertrophied lenticels, adventitious roots, and aerenchyma, to enhance ventilation of the submerged organs to avoid the energetic crisis caused by oxygen shortage (Fukao et al., 2019).

In the post-flooding period, several responses were the exact opposite to those during flooding. Most growth variables were higher or similar in F40; while height was significantly lower in F40 than in F10. A possible explanation could be that in the immediate aftermath of flooding, all aquatic roots in F40 dried down (Figure 2B), dramatically reducing the root-to-shoot ratio. At the end of the post-flooding period the root-to-shoot ratio was still significantly lower in F40 compared with Control and F10 plants. It is likely that, in the post-flooding period, F40 plants were investing resources in developing new roots to compensate for the losses, instead of increasing the growth in height. This is not only evident in roots, as F40 plants had a higher leaf area and GRD. Clearly, there was a different assignation of growth resources for F10 and F40 in the post-flooding period.

### Development of Vessels and Hydraulic Conductivity

Xylem hydraulic conductivity, and vessels number and size are plastic traits regarding flooding. Herrera et al. (2008b) reported that hydraulic conductivity was reduced in early stages of flooding, but increased later with the development of adventitious roots in tropical flood-tolerant tree species. Partial flooding reduced vessel size in stems of *Quercus robur* (Copini et al., 2016) and in two willow genotypes (Doffo et al., 2017).

We found a sharp contrast in the development of xylem vessels in both treatments. During flooding, F40 produced less vessels of similar area than the Control treatment. In the post-flooding period, it was exactly the opposite: F40 produced fewer vessels with a higher area than Control plants. If we consider the fraction of the lumen area (i.e., the actual water conducting area of the stem), the conducting area was mostly produced during flooding in the plants of Control and F10, but during post-flooding in F40. The plants from F40 compensated the reduction in conducting area producing few bigger vessels in the post-flooding period. Eventually, lumen area was similar in all treatments, which explains the similar values of xylem hydraulic conductivity observed in them (Supplementary Figure 4). The increase in vessels size and stem diameter may be associated with the need to increase water transport to supply the higher leaf area developed by F40 in the post-flooding period.

These results are similar to those described for *Quercus robur* (Copini et al., 2016), where flooding reduced early wood vessels size in the submerged parts of the stem (as LF in F40) but not in the non-submerged parts (as LF in Control and F10 plants). This comparison should be considered with caution for the anatomical differences between the two species. Oaks are ring porous species, with big early wood vessels that account for an increased hydraulic conductivity in early season, while willows are diffuse porous species, producing vessels of similar size throughout the growing season (Crang et al., 2018). Anyway, it is interesting to note that flooding of the stem seems to induce the same response in oaks and willows, in spite of the anatomical and functional seasonal differences in the xylem between the two species. The results for F10 are different from the reported in Doffo et al. (2017), where 2 willow genotypes flooded 10 cm above soil level for 45 days had a lower vessel area than Control plants. These differences could be due to the different length of the flooding period, or caused by genetic factors since the genotypes used in that work were different (*S. alba* and a *S. matsudana × S. alba* hybrid).

### Flooding Depth Effects on Photosynthetic Activity and Other Related Leaf Traits

The acclimation responses to flooding of the photosynthetic machinery are already known. For instance, gray poplar and oak decreased their photosynthetic rate and increased leaf protein content under root flooding, but the chlorophyll content remained unchanged (Kreuzwieser et al., 2002). In several tropical species that acclimate to long-term flooding, there was an increase in leaf protein and chlorophyll content (Herrera, 2014). In some willow genotypes, flooding increased specific leaf area and nitrogen content of leaves, but others did not (Rodríguez et al., 2018). In consequence, we were interested in exploring how changes caused by flooding on leaves affected the photosynthetic responses during post-flooding. The leaves that developed during flooding may have different morphological and biochemical traits that may affect their photosynthetic activity. For this reason, we tagged and compared leaves expanded during flooding and post-flooding periods. The photosynthetic rate was higher in leaves that developed during flooding and post-flooding in F40, when compared to Control treatment. This may partly be the result of an increase in leaves nitrogen content during flooding that persisted into the post-flooding period. More nitrogen implied higher chlorophyll content (and likely leaf protein as well), these traits had shown a strong positive correlation with photosynthesis in a set of 11 willow genotypes (Andralojc et al., 2014). The higher photosynthetic rate in F40 plants may also be the result of an enhanced stomatal conductance. Stomatal conductance increased in F10 plants, and this is likely the reason of the increase in carbon isotopic discrimination, and the decrease in water use efficiency for this treatment. The higher stomatal conductance in previously flooded plants could not be totally accounted for by an increased stomatal density, because the differences between treatments were not always statistically significant. The chlorophyll a/b ratio did not change during flooding or post-flooding periods (data not shown); these results were different from flooded poplar and beech where the ratio increased (Kreuzwieser et al., 2002). During flooding, the deeper flooded plants increased specific leaf area and leaf nitrogen, as described before (Rodríguez et al., 2018), and this higher content was maintained during the post-flooding period for the genotype we used in this study.

Consistently with the higher photosynthetic activity, the F40 leaves showed higher soluble sugar content, but did not increased the non-soluble (starch) sugars. Starch accumulation in leaves has been related to the inhibition of photosynthesis (Rengifo et al., 2005; Andralojc et al., 2014) and clearly this is not the case in previously flooded F40 plants. This scenario is consistent with these leaves producing and exporting more photosynthates to other organs.

### Different Responses to Floodwater Depth During Flooding and Post-flooding in Willows

**FIGURE 7:**
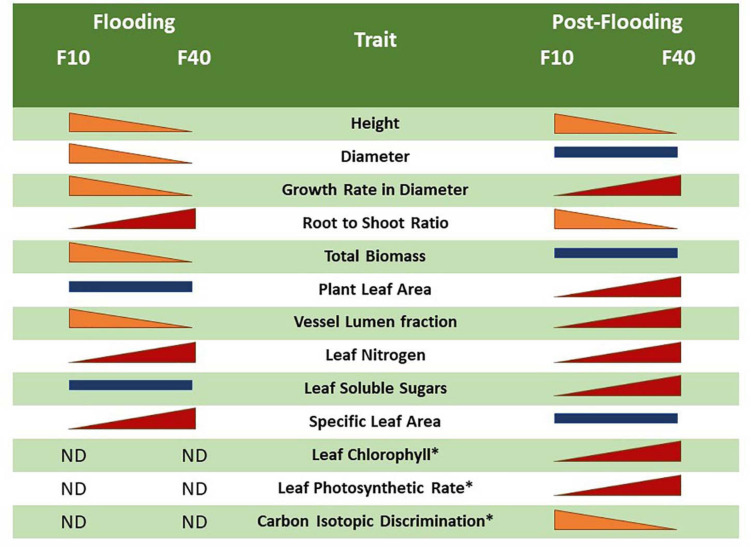
Comparison between the flooding and post-flooding responses among the treatments F10 (plants flooded 10 cm above soil level) and F40 (plants flooded 40 cm above soil level). The change of size of the bars indicates whether a trait increased or diminished compared to the other flooding treatment. Bars of similar size indicates no significant differences between the flooding treatments. The symbol “*” represents an average of L1 and L2 leaves.

The differences in flooding and post-flooding responses between F10 and F40 treatments are summarized in Figure 7. The F40 plants experienced growth reduction in height, diameter and biomass during flooding, but they showed an increased compensatory growth in diameter during post-flooding. This extra growth was sustained by the increased photosynthetic activity, very likely due to higher leaf chlorophyll, nitrogen and stomatal conductance. F40 plants had higher photosynthesis per plant, because they increased leaf area development during post-flooding (Supplementary Figure 5). The bigger leaf area is sustained by an increased stem growth in diameter, and the development of xylem vessels with a greater area to compensate for the diminished vessel lumen fraction during flooding. In summary, the plants under deeper floodwater experienced higher growth reduction than the shallow flooded plants, but they showed compensatory responses during post-flooding that made up for the previous losses. Even when plants are only partially flooded, floodwater depth induced different physiological responses that persisted after the end of the flooding episode. In this sense, willow responses are different from *Populus deltoides*, where partial flooding led to a plant leaf area increase in the post-flooding period, but without an increment of the photosynthetic activity per leaf area (Rodríguez et al., 2015). The plastic responses of willow after the stress episode are similar to the growth compensatory responses of young trees of other pioneer riparian species, *Populus fremontii*, *Tamarix ramosisiima*, and *Acer negundo* (Kui and Stella, 2016). For instance, seedlings that survived partial sediment burial increased their growth in the following season (Kui and Stella, 2016).

## Conclusion and Perspectives

Our first hypothesis was confirmed, since the physiological responses varied during post-flooding according to floodwater depth. Once the flooding episode ended, the deep flooded plants (F40) compensate the greater growth reduction compared with the shallow flooded treatment (F10) by increasing photosynthetic activity, leaf area and xylem lumen fraction in the post-flooding period.

The second hypothesis was also accepted as morphological and biochemical differences were observed between the leaves expanded during and after the flooding episode.

Our results underscore the fact that flooding tolerance of willows is not only caused by the responses during the occurrence of the stress, but also by the compensatory photosynthetic rate and differential growth of organs during post-flooding. Willow resilience is determined by their plasticity to respond to the challenges during the post-flooding period, in addition to what happens during the stress period itself.

## Data Availability Statement

The raw data supporting the conclusions of this article will be made available by the authors, without undue reservation.

## Author Contributions

IM did most experimental work and the statistics. MR collaborated on the experimental work. SM performed the xylem anatomical study and planned the experimental work. VL planned the experiment and wrote the manuscript. All the authors have read and approved the manuscript.

## Conflict of Interest

The authors declare that the research was conducted in the absence of any commercial or financial relationships that could be construed as a potential conflict of interest.
