# Supplementary material for: Floodwater Depth Causes Different Physiological Responses During Post-flooding in Willows
Source: Front Plant Sci. 2021 May 21;12:575090. doi: 10.3389/fpls.2021.575090 (PMC8176222; doi:10.3389/fpls.2021.575090)
Supplement: Supplementary file 1 [file Data_Sheet_1.PDF]

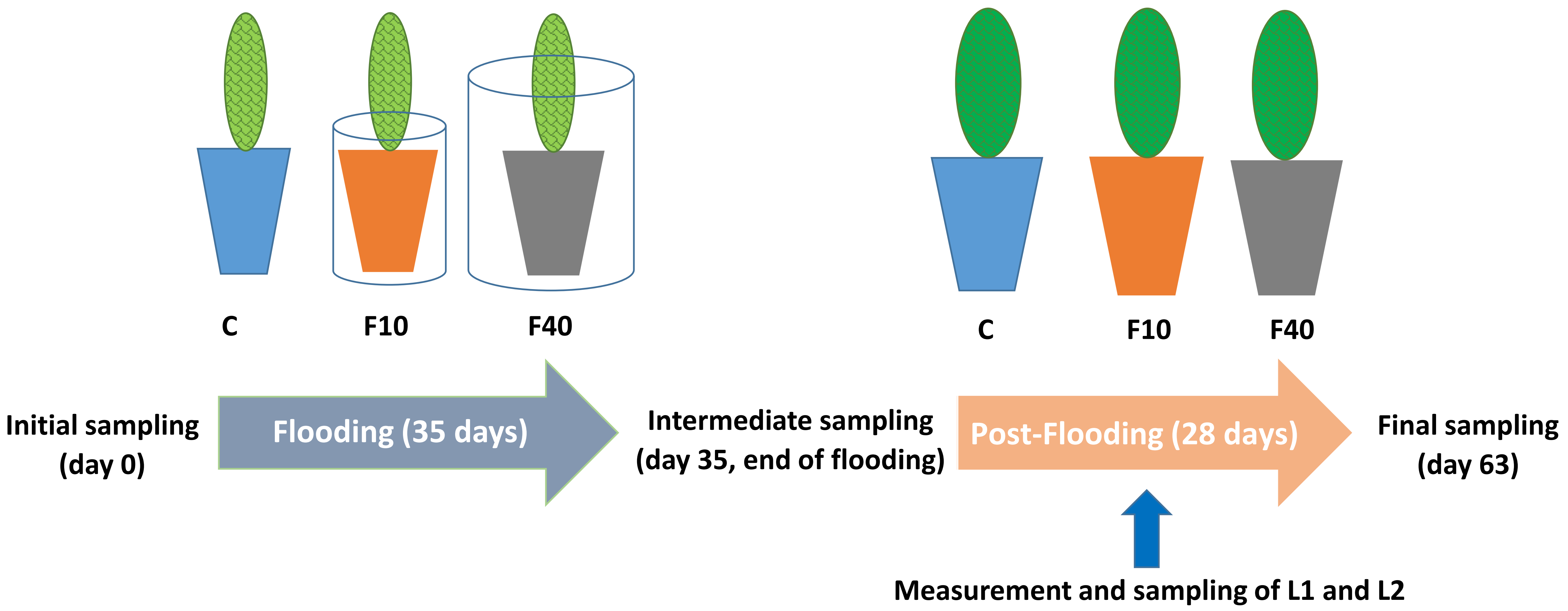

S. Fig. 1. An outline of the experiment. C: plants watered to field capacity. F10: plants flooded 10 cm above soil level. F40: plants flooded 40 cm above soil level. L1: leaf expanded during flooding. L2 : leaf expanded during the post-flooding period.

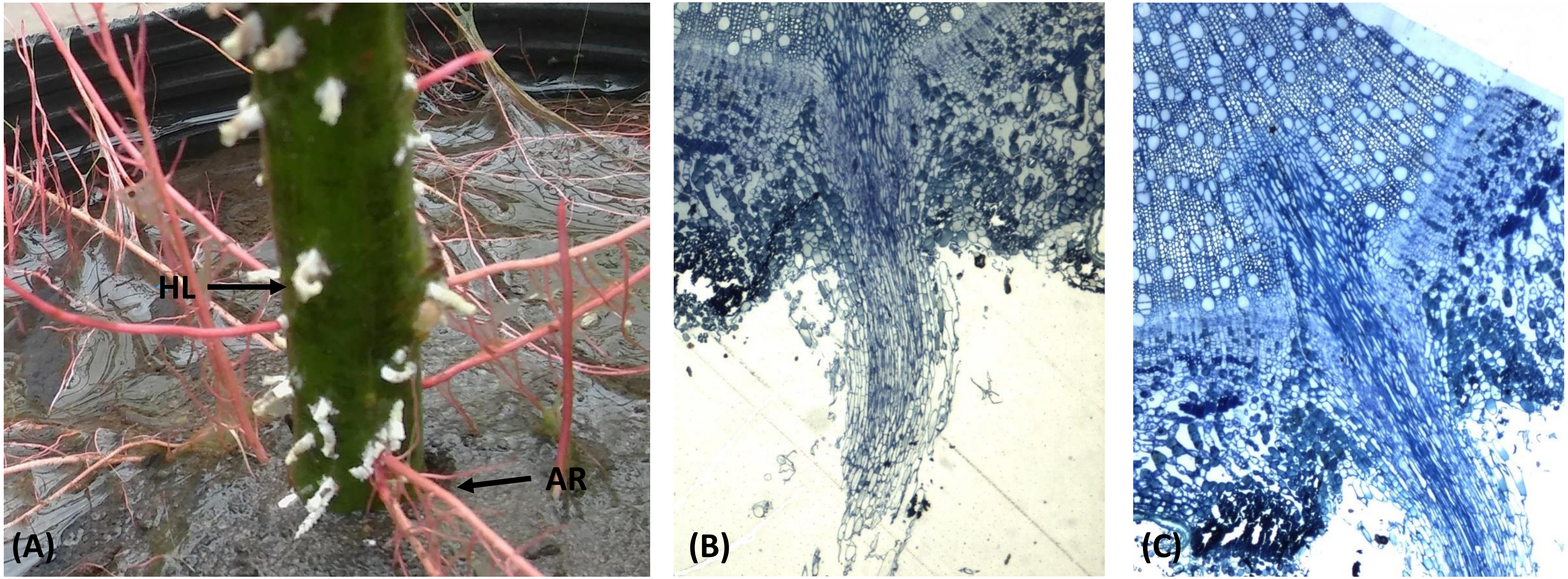

S. Fig. 2. Hyperthrophied lenticels (HD) and aquatic roots (AR) developing on the stem of an F40 plant removed from wáter (A); histology of a hyperthrophied lenticel developed in the stem of a F40 plant (B and C).

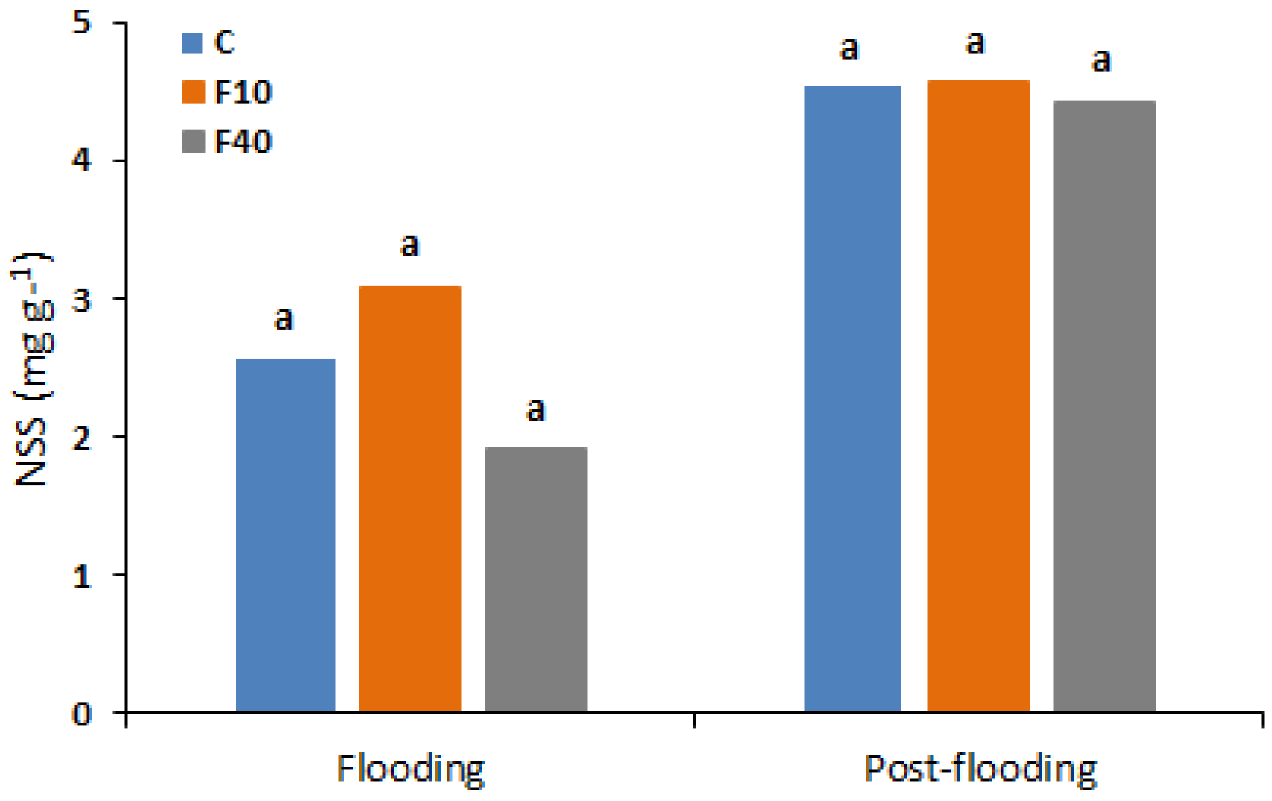

S.Fig.3. Non- soluble sugars (NSS) measured at the end of the flooding (day 35) and post-flooding period (day 63). Means followed by the same letter did not differ significantly (Tukey $p<0.05$).

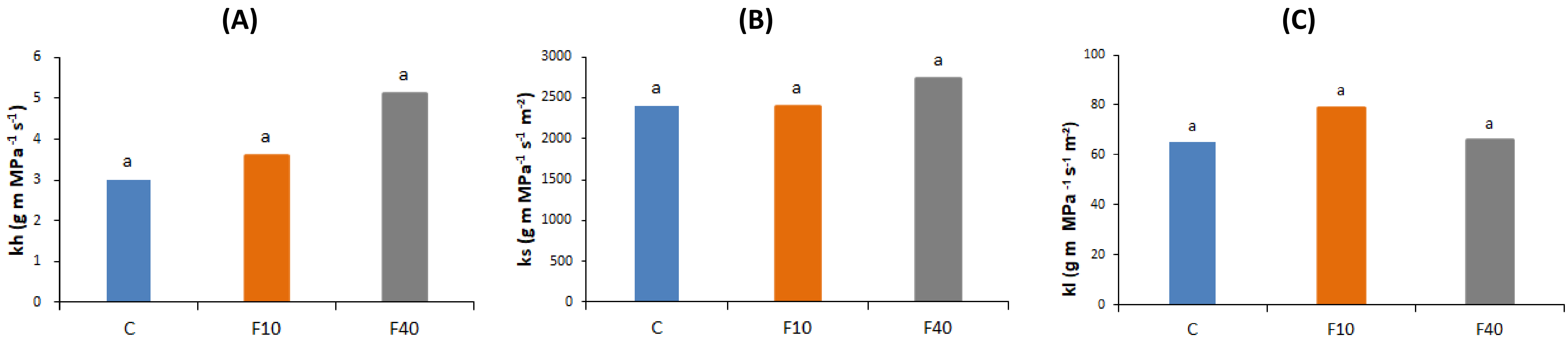

S.Fig.4. Hydraulic conductivity per unit stem length (kh, panel A), hydraulic conductivity per unit xylem area (ks, panel B) and hydraulic conductivity per unit leaf area (kl, panel C), measured at the end of the post-flooding period (day 63). Means followed by the same letter did not differ significantly (Tukey $p<0.05$).

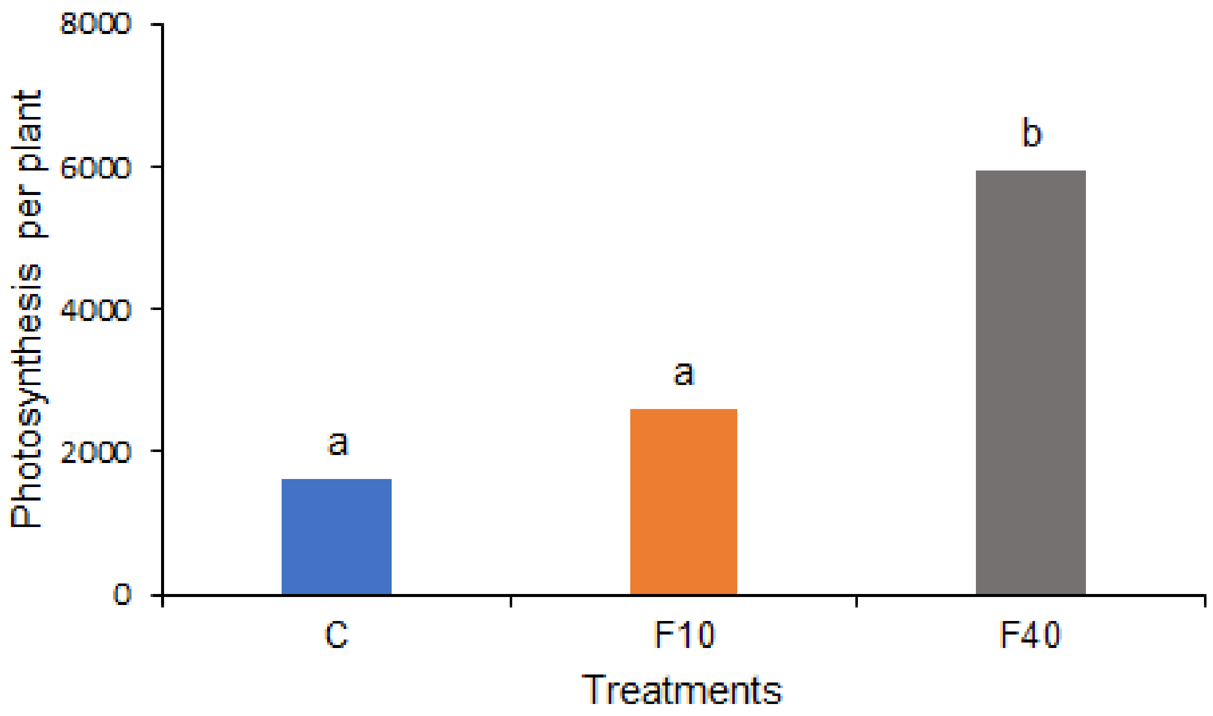

S.Fig.5. Photosynthesis per plant by leaves developed in the post-flooding period. Means followed by the same letter did not differ significantly (Tukey $p<0.05$). The estimation was made multiplying the leaf area by L2 leaf photosynthetic rate.
